# Influence of a Selenium Biofortification on Antioxidant Properties and Phenolic Compounds of Apples (*Malus domestica*)

**DOI:** 10.3390/antiox9020187

**Published:** 2020-02-24

**Authors:** Sabrina Groth, Christoph Budke, Susanne Neugart, Sofia Ackermann, Fenja-Sarah Kappenstein, Diemo Daum, Sascha Rohn

**Affiliations:** 1 Hamburg School of Food Science, Institute of Food Chemistry, University of Hamburg, Grindelallee 117, 20146 Hamburg, Germany; sabrina.groth@chemie.uni-hamburg.de (S.G.); ackermannsofia@aol.com (S.A.); nudel5555@web.de (F.-S.K.); 2 Department of Plant Nutrition, Osnabrück University of Applied Sciences, 49090 Osnabrück, Germany; c.budke@hs-osnabrueck.de (C.B.);; 3 Department of Crop Sciences, Division Quality and Sensory of Plant Products, Georg-August-Universität Göttingen, 37075 Göttingen, Germany; susanne.neugart@uni-goettingen.de

**Keywords:** apple, selenium, agronomic biofortification, antioxidant activity, phenolic compounds, TEAC, Total Phenolic Content, phenoloxidase

## Abstract

Biofortified apples seem to be a suitable produce. In this study, different selenium forms and application levels were applied to the two apple varieties ‘Golden Delicious’ and ‘Jonagold’, grown in the years 2017 and 2018 in order to increase the selenium uptake within a typical Western diet. It was shown that the biofortification, which was performed as a foliar application implemented in usual calcium fertilization, led to significantly increased selenium contents in the fruits. Furthermore, biofortification affected the total phenolic content (TPC), the polyphenol oxidase activity (PPO), as well as the antioxidant activity (AOA), the latter measured with the two well-known assays Trolox Equivalent Antioxidant Capacity Assay (TEAC) and Oxygen Radical Absorbance Capacity Assays (ORAC). The varying selenium forms and application levels showed a differing influence on the parameters mentioned before. Higher fertilizer levels resulted in higher selenium accumulation. It was found that PPO activity fluctuates less in biofortified apples. With regard to TPC, selenate led to higher amounts when compared to the untreated controls and selenite resulted in lower TPC. AOA analysis showed no clear tendencies as a result of the selenium biofortification. In the case of ‘Jonagold’, a higher AOA was generally measured when being biofortified, whereas, in the case of ‘Golden Delicious’, only one form of application led to higher AOA. Additionally, differences in the amount of major phenolic compounds, measured with High Performance Liquid Chromatography Mass Spectrometry (HPLC-DAD-ESI-MS^n^), were observed, depending on the conditions of the biofortification and the variety.

## 1. Introduction

Biofortification is an agronomic practice for specifically enriching food crops with certain nutrients. In most cases, it is aimed at increasing the content of minerals, such as zinc or selenium, because soil conditions often do not allow for a natural presence of adequate amounts of these compounds [1,2,3,4]. Especially in Germany and other European regions, selenium is only present in small amounts in the soils, which means that the selenium content of plant produce is correspondingly low [5]. As a result of targeted applications of selenium-containing fertilizers, the plant increasingly absorbs the mineral, which is then integrated into the endogenous plant metabolism by incorporation into amino acids, such as selenocysteine and selenomethionine [6].

Since 1985, foods, such as cereals, have been successfully biofortified with selenium in Finland. There, it was shown that the selenium supply of the mean population steadily improved [7]. Biofortification seems to be suitable for addressing selenium deficiency that many people are suffering from worldwide. Consequently, selenium deficiency-related diseases, such as reduced immune function, degeneration of the cardiovascular system, and cognitive decline, could be minimized [4,8,9]. A prolonged deficiency of selenium is associated with the endemic diseases Keshan and Keshin-Beck [9].

Selenium is an essential element in human nutrition and therefore plays an important role in the human organism, especially as a component of proteins and enzymes such as glutathione peroxidase, thyroxine 5-deiodinase, and selenoprotein P [9]. Selenium is involved in the production of active thyroid hormones and the regulation of the immune system. It is essential for reproduction and has antioxidant, anti-inflammatory, and antiviral effects [10]. Furthermore, selenium is an integral part of some antioxidant enzymes, which protect cells from being damaged by radicals produced during oxidative metabolic pathways [9]. Some benefits of higher selenium status on the risk of prostate, lung, colorectal, and bladder cancers have already been described [8].

The recommendations for the daily selenium intake for Germany, Austria, and Switzerland (D-A-CH reference values for nutritional intake) are approximately 1 µg selenium per kg body weight. With consideration of the reference body weights, the resulting estimated values for selenium intake are approximately 70 µg/day for adult men and 60 µg/day for adult women [11]. Usually, the need for selenium is largely covered by animal produce, such as meat or fish. The biofortification of plant produce allows for vegetarians or vegans in particular to fulfill their needs naturally and as an alternative to food supplements [12,13]. A previous consumer survey clearly indicated that German consumers would prefer selenium-rich apples instead of food supplements for improving their selenium supply [14]. Another advantage of the intake of selenium-containing food as compared to the intake of supplements is the different bioavailability of the varying selenium forms. The organic forms (when anorganic selenium has been already transformed to organic compounds, such as selenocysteine and selenomethionine in plants) can be more easily absorbed in the intestinal tract when compared to the inorganic forms, being often present in dietary supplementation products [9,10].

In Germany, apple is the most popular fruit with a consumption of 21.0 kg per capita [15] and therefore particularly suitable for reaching a broad proportion of the population with a chance for improving the selenium supply for many people.

Other research groups already carried out studies on various crops for selenium biofortification, while using foliar fertilization with sodium selenite or sodium selenate. It has been shown that those treatments led to increased selenium levels in the plants when compared to the untreated controls. A higher accumulation with selenate was observed when compared to selenite [16,17,18,19]. Furthermore, the influence of different fertilizer levels was investigated, where it was observed that the selenium content in the plants increased with increasing selenium level [16,18,19,20,21,22]. However, Hawrylak-Nowak found a decline in the biomass of hydroponically cultivated butterhead lettuce (*L. sativa* L. var. *capitata*, cv. ‘Justyna’) at the highest tested level of 15 µM Se that was contained in the nutrient solution [20].

In addition to the selenium content, the influence of a biofortification on other parameters, such as fruit quality, and the content of secondary plant substances, such as phenolic compounds, were also investigated in several studies: Zhao et al. found a significant increase of vitamin C content in selenium biofortified pear-jujube (*Ziziphus jujuba* M. cv. ‘Lizao’) [23]. D’Amato et al. conducted a study on olive oil in which significantly higher levels of phenolic compounds and changes in the phenolic profile were observed, with the contents of certain antioxidant phenolic compounds increasing, as a result of the biofortification [24]. Further analyses on rice (*Oryza sativa* L., cv. ‘Selenio’) showed that moderate doses of selenite up to 45 mg/L were the best compromise between high selenium levels and an increase of phenolic acid concentration [16]. Schiavon et al. also found elevated phenolic compound contents in tomatoes (*Solanum lycopersicum* L., cv. ‘Margoble’), resulting from a selenium biofortification [21]. Especially, they found elevated levels of the antioxidant flavonoids naringenin chalcone, and kaempferol. However, a simultaneous decrease of cinnamic acid derivatives was observed [21]. In a subsequent study, the same research group investigated the influence of a selenium biofortification on the leaves and roots of radish (*Raphanus sativus* L., cv. ‘Saxa’). The total phenolic content (TPC) in the roots was reduced by 40–60%, whereas an increase of 10% of the TPC was observed in the leaves when compared to leaves of the control plants [22]. Bachiega et al. investigated the relationship between phenolic compounds and the antioxidant activity (AOA) in selenium biofortified broccoli (*Brassica oleracea*, cv. ‘Italica’). They found a significant increase in phenolic compounds as well as a higher AOA [25]. Pezzarossa et al. performed studies on peaches (*Prunus persica*) and pears (*Pyrus communis* L.) that were biofortified with sodium selenite: There, an extended shelf life of the fruits after removal from the storage was shown, being hypothesized to be related to increased TPC [26].

So far, only few data exist on the selenium biofortification of apples. In 2019, Babalar et al. investigated the influence of a selenium biofortification with sodium selenate on the apple variety ‘Starking Delicious’ and various quality parameters, also including the TPC. However, it was only analyzed as a function of the storage time of the fruit; a direct comparison between biofortified and untreated apples was not done [27]. The aims of the present study were to increase the selenium concentration in the apples and identify the appropriate dosage form and application level. A further focus was on the increase of value-added phytochemicals, especially on substances related to AOA. The relationship between selenium biofortification with phenolic compounds and antioxidant properties in apples has not yet been investigated. Within the scope of this work, this was analyzed at hand of a large number of different applications, in which the selenium form and the level of application were varied. The two apple varieties ‘Golden Delicious’ and ‘Jonagold’ were studied in two consecutive years because of being very important cultivars for the German market.

## 2. Materials and Methods

### 2.1. Chemicals

Disodium hydrogen phosphate dodecahydrate was purchased from Bernd Kraft GmbH (Duisburg, Germany). Sodium dihydrogen phosphate monohydrate was from AppliChem GmbH (Darmstadt, Germany) and catechol was from ThermoFisher GmbH (Kandel, Germany). Aceton was used from VWR International LLC (Fontenay-sous-Bois, France). The standards for the HPLC analysis (chlorogenic acid, catechin, epicatechin, phloretin-2-glucoside, and quercetin 3-glucoside) and hydrochloric acid (25%) were from Carl Roth GmbH & Co. KG (Karlsruhe, Germany). Sodium carbonate was purchased from Grüssing GmbH (Filsum, Germany) and potassium peroxodisulphate was from Fisher Scientific UK Ltd. (Loughborough, UK). Folin-Ciocalteu’s phenol reagent, potassium dihydrogen phosphate, and nitric acid (65%) were purchased from Merck KgaA (Darmstadt, Germany). Galllic acid and 2,2’-azobis(2-methylpropionamidine) dihydrochloride were from Fisher Scientific GmbH (Schwerte, Germany). 2,2’-Azino-bis-(3-ethylbenzthiazoline-6-sulfonic acid) diammonium salt, trolox, and fluorescein were purchased from Sigma-Aldrich Chemie GmbH (Deisenhofen, Germany). All of the chemicals were of analytical grade. Water was purified through a Milli-Q water system (PURELAB^®^, Elga LabWater, Veolia Water Technologies GmbH, Celle, Germany) and was used for buffers, the extracting agents, and dilution of sample extracts.

### 2.2. Sample Material

The apple cultivars ‘Golden Delicious’ and ‘Jonagold’ were evaluated. Fruits were grown in 2017 and 2018 at the Horticultural Research Station of the Osnabrück University of Applied Sciences, Germany (N52.310654°, E008.02844°; 69 m a.s.l.).

The apple trees were grown on Plaggen soil, the topsoil, and subsoil were loamy sand. The pH value of the soil was 5.5 and the organic matter content in the topsoil amounted 2.4% and in the subsoil 1.8%. On an adjacent, the horticulturally used area (also loamy sand), the soil Se content determined by extraction with aqua regia amounted to 0.25 mg/kg d.w. and by extraction with 0.1 M K_2_HPO_4_/KH_2_PO_4_ (pH 7.0) was below the detection limit (< 0.1 mg/kg d.w.). This indicates that a relatively low content of phytoavailable Se was present at the test site. The very low Se contents of the untreated control apples that ranged between 0.2 and 0.4 µg Se/100 g d.w. confirm that the selenium content in the soil was very low.

The experimental plant was a randomized block plant design with four repetitions, whereby one tree corresponded to one repetition. The trees were treated and harvested from both sides. The rows were aligned in north-south direction. The apples were biofortified with selenium while using a foliar fertilization approach and the selenium forms sodium selenate and sodium selenite were used in analytical grade. Foliar fertilization is advantageous over soil application, as plants are directly treated and, thus, compounds might easily enter fruits. Only the selenium that reaches the fruit is relevant for biofortification, since selenium is not significantly shifted from the leaves into the fruit. Furthermore, application can be done in parallel with the traditionally performed calcium sprays [28]. For improving wetting properties, all of the solutions used for foliar sprays additionally contained 0.02% (*v*/*v*) of the nonionic organosilicone adjuvant Break-Thru^®^ S 240 (AlzChem AG, Trostberg, Germany).

In 2017, the apples were biofortified with 0.15 kg Se per hectare and meter canopy height (Se/ha x m CH), divided into six applications during the season (beginning in July until the end of September) with a hand-held spray system (model Easy-Sprayer Plus, Lehnartz GmbH, Remscheid, Germany). Pure water was sprayed on the trees for the control treatments. During sampling, ten well-developed medium size apples from well exposed middle parts of the trees were harvested for subsequent analysis.

The apples were processed at the Osnabrück University of Applied Sciences after two weeks of storage at 2 °C. First, the fruits were divided into eight segments and the stalk while using an apple slicer. The stalk segment was discarded. The segment samples were directly shock frozen with liquid nitrogen and stored at −27 °C.

In 2018, the application rate of the fertilizer was reduced to 0.075 kg Se/ha x m CH and only sodium selenate was applied. The application rate was reduced in 2018 due to slight fruit damages occurring in the year 2017. The selenium fertilizer was applied together with the calcium-containing foliar fertilizer WUXAL^®^Ascofol Ca (5 L/ha; Aglukon Spezialdünger GmbH &Co. KG, Düsseldorf, Germany). For the control treatments, pure water and WUXAL^®^Ascofol Ca were sprayed on the trees. In that season (end of June until the end of August), a backpack sprayer (REB 15 AZ2, Birchmeier Sprühtechnik AG, Stetten, Switzerland) was used for application. Sampling was analogous to the previous year.

With the exception of the enzyme activity determination of the polyphenol oxidase, which was done from thawed apple samples, all other samples were lyophilized to prevent the degradation of the phenolic compounds. A frozen sample was placed in a knife mill (Blixer^®^ 4-3000; robot coupe S.N.C., Vincennes Cedex, France) with the addition of dry ice and homogenized for 60 s at 3000 rpm. After homogenization, the sample was freeze dried for 48 h. The dried samples were then filled into 50 mL tubes and then stored at −20 °C until further analysis. A sample set of four randomly chosen apples per variety were analyzed.

### 2.3. Determination of the Selenium Content

For the determination of the selenium content, the apples were pre-prepared at the Osnabrück University of Applied Sciences, as described in 2.2. After the subdivision into eight segments, the samples were directly dried at 60 °C in a fresh air drying oven until their weight remained constant. After drying, the samples were ground in an ultracentrifugal mill (Retsch ZM 200, Retsch GmbH, Haan, Germany) at 14,000 rpm to a particle size ≤ 0.5 mm. The powder was stored in plastic tubes until further sample preparation. A sample digestion was carried out according to the standardized method DIN EN 13805 [29]. For this purpose, 0.5 g of the ground plant material was digested while using microwave pressure digestion in quartz glass vessels with 65% nitric acid at 190 °C. The digestion solution was measured with a graphite tube atomic absorption spectrometer (Thermo Scientific UNICAM SOLAAR M Series AA, Thermo Fisher Scientific Inc., Waltham, MA, USA). Internal and external certified reference material was used to ensure the quality of the analysis [ERM-BB422 fish muscle and NIST-1849a infant/adult nutritional (milk) powder]. For samples with low selenium concentrations (< 2.5 µg/L), selenium analysis was alternatively carried out while using the hydride technique in accordance with DIN 38405-23 [30].

### 2.4. Determination of the Polyphenol Oxidase (PPO) Activity

The determination of the PPO activity of the apple samples was done according to Kolodziejczyk et al. [31] and González et al. [32], with an adaption to a miniaturized procedure. About 10 g of the frozen sample was weighed and crushed in a mortar. Subsequently, 25 mL of a phosphate buffer (0.05 M, pH 7.0) were added and then mixed. The sample was incubated for 120 min. at 4 °C in the dark, centrifuged (15 min., 4 °C, 3225 g), and the supernatant used to determine PPO activity. First, 30 µL of the sample extract were given in a 96-well microtiter plate and either 270 µL of the phosphate buffer (0.2 M, pH 5.5) as blank value or 270 µL of a catechol solution (0.1 M in 0,2 M phosphate buffer, pH 5.5) were added. The enzyme activity was immediately determined at 25 °C by measuring the change in absorption over 10 min. at a wavelength of λ = 420 nm with a BioTek Synergy HT microplatereader (BioTek Instruments Inc., Winooski, VT, USA), whereby the change in absorption was recorded every 60 s. The enzyme activity was given as activity units per 100 g of fresh weight (f.w.) of a fruit sample. One unit is defined as the change of 0.01 in the absorbance value per minute [31,32].

### 2.5. Method for Extracting Phenolic Compounds

Sixty miligrams of the lyophilized apple sample were weighed into a 2 mL tube. One milliliter of the extraction agent 50% aqueous acetone and 0.1% HCl (v/v) was added and treated in an ultrasonic bath for 5 min. at 30 °C. Subsequently, four glass beads (i.D. 4 ± 0.3 mm) were added to each sample and the sample was ground and mixed in a ball mill (5 min., 25 Hz). The samples were then centrifuged for 5 min. at 20,817 g and the supernatant transferred into a 15 mL tube. The extraction with the ball mill was repeated twice and supernatants were combined. The total volume was filled to 4 mL.

### 2.6. Determination of the Total Phenolic Content (TPC) according to Folin-Ciocalteu

The TPC of the apple samples was determined while using a modified Folin-Ciocalteu methodology, according to Müller et al. [33]. Twenty microliters of the sample extract were given in a 96-well microtiter plate, 100 µL of the Folin-Ciocalteu phenol reagent (1:10; *v*/*v*), and 80 µL of an aqueous 7.5% (*w*/*v*) sodium carbonate solution were added. Subsequently, incubation was performed for 2 h at room temperature in the dark. The absorption was measured at a wavelength of 765 nm at 30 °C while using the BioTek Synergy HT microplatereader. TPC is given in gallic acid equivalents per 100 g of dry weight (mg GA/100 g d.w.) [33,34].

### 2.7. Analysis of the Antioxidant Activity (AOA) using the Trolox Equivalent Antioxidant Capacity Assay (TEAC)

The determination of the AOA using the TEAC assay was performed according to Müller et al. [33]. A 75 mM phosphate buffer (pH 7.4) as well as a 7 mM 2,2’-Azino-bis(3-ethylbenzothiazoline-6-sulfonic acid) diammonium salt (ABTS) stock solution and a 2.45 mM potassium peroxodisulphate solution were prepared. Both of the solutions were mixed, transferred to an amber glass bottle, and stored for 24 h at room temperature, until the ABTS·^+^ radical was completely formed. The reagent, known as ABTS working solution I, was then stored in a refrigerator. Two hours before starting a determination, the ABTS working solution I was diluted with phosphate buffer (75 mM, pH 7.4) to an absorbance of E_730_ = 0.700 ± 0.050. This ABTS working solution II was left at room temperature until measurement. For calibration, a 2.5 mM trolox stock solution was prepared and diluted 1:10 (*v*/*v*) with water. A dilution series was prepared from this. Twenty microliters of various dilutions of the samples, trolox, or water (blank value) were given in a 96-well microtiter plate and 200 µL of ABTS working solution II were added. The adsorption was measured after 6 min. incubation at 30 °C at a wavelength of λ = 730 nm with the BioTek Syngergy HT microplatereader. AOA is calculated as trolox equivalent 100 g dry weight per (mmol TE/100 g d.w.) [33,35].

### 2.8. Analysis of the AOA using the Oxygen Radical Absorbance Capacity Assays (ORAC)

For the ORAC assay, which was also done according to Müller et al. [33], a 0.12 mM fluorescein solution was prepared from fluorescein and phosphate buffer (75 mM, pH 7.4). From this solution, the final fluorescein working solution was freshly prepared by a 1:100 dilution with phosphate buffer, before each analysis. For the 2,2’-azobis(2-methylpropionamidine) dihydrochloride (AAPH) stock solution (c = 129 mM), AAPH was dissolved in phosphate buffer. Calibration was also done with trolox. Ten microliters of each sample in the different dilutions, trolox, or water were given in a 96-well microtiter plate. Subsequently, 100 µL phosphate buffer (75 mM, pH 7.4) or 250 µL for the negative control were added. After a 10 min. incubation period in the BioTek Synergy HT microplatereader at 37 °C, 150 µL of the AAPH stock solution were added to the blank value, standards, and samples. The measurement, which was based on fluorescence quenching, was performed at an excitation wavelength of λ = 485 nm and an emission wavelength of λ = 528 nm at 37 °C. The course of the reaction was recorded for 120 min., with one measurement every two minutes. AOA is also calculated as trolox equivalent per 100 g dry weight (mmol TE/100 g d.w.) [33,36].

### 2.9. Qualitative and Quantitative Determination of Phenolic Compounds Using High Performance Liquid Chromatography Mass Spectrometry (HPLC-MS)

The phenolic compounds were extracted from the lyophilized apple samples (0.01 g) in a triple extraction with 60% aqueous methanol, according to Neugart et al. [37]. Phenolic compound identification and quantification were determined while using an 1100 series HPLC system (Agilent Technologies GmbH, Waldbronn, Germany) equipped with a degasser, binary pump, autosampler, column oven, and photodiode array detector. An Ascentis^®^ Express F5 column (150 mm × 4.6 mm, 5 µm, Sigma-Aldrich Chemical Co., St. Louis, USA) was used to separate the compounds at a temperature of 25 °C. Eluent A was 0.5% acetic acid, and eluent B was 100% acetonitrile. The gradient used for eluent B was 5–12% (0–3 min.), 12–25% (3–46 min.), 25–90% (46–49.5 min.), 90% isocratic (49.5–52 min.), 90–5% (52–52.7 min.), and 5% isocratic (52.7–59 min.). The determination was conducted at a flow rate of 0.85 mL/min. and wavelengths of 280 nm, 320 nm, and 370 nm for phloretin glycosides and flavanols, hydroxycinnamic acid derivatives, and non-acylated flavonol glycosides, respectively. The hydroxycinnamic acid derivatives and glycosides of flavonols were identified as deprotonated molecular ions and characteristic mass fragment ions according to Schmidt et al. [38] by HPLC-DAD-ESI-MS^n^ while using an Agilent ion trap mass spectrometer in negative ionization mode. Nitrogen was used as the dry gas (10 L/min, 325 °C) and the nebulizer gas (40 psi) with a capillary voltage of −3500 V. Helium was used as the collision gas in the ion trap. The mass optimization for the ion optics of the mass spectrometer for quercetin was performed at *m/z* 301 or arbitrarily at *m/z* 1000. The MS^n^ experiments were performed up to HPLC-DAD-ESI-MS^3^ in a scan mode from *m/z* 200–2000. The standards (chlorogenic acid, catechin, epicatechin, phloretin-2-*O*-glucoside, and quercetin-3-*O*-glucoside*)* were used for external calibration curves. The results are presented as mg/100 g dry weight.

### 2.10. Statistical Analysis

The number of analyses per application with selenium fertilizer or control was *n* = 4. All of the analyses were done twice. The data are given in mean ± standard deviation and further evaluated while using Microsoft Excel (Microsoft Office Professional Plus 2016, Redmond, WA, USA). The statistical analyses were carried out using SPSS (Version 25, IBM^®^ Corporation, Armonk, NY, USA) and the data were further evaluated with a two-way analysis of variance (ANOVA). The means were compared while using the Bonferroni post-hoc test at *p* < 0.05.

## 3. Results and Discussion

Table 1 gives an overview of the results of the determination of the selenium content, the polyphenol oxidase activity, the total phenolic content, and the antioxidant activity that was determined with both assays - TEAC and ORAC in the biofortified apples of the varieties ‘Golden Delicious’ and ‘Jonagold’ with the various selenium applications, as well as the corresponding untreated controls of the years 2017 and 2018.

### 3.1. Selenium Content

Biofortification significantly increased in the selenium content of apples in general as compared to the untreated controls (Table 1). This increase was 10 to 14-fold. The highest content was achieved in both varieties, which was 5.6 µg Se/100 g f.w when applying 0.15 kg Se per hectare and meter canopy height in the form of selenite in 2017. The application of selenate at the same dosage level also led to an identical Se level in ‘Golden Delicious’ and a slightly lower content of 4.5 µg Se/100 g f.w. in ‘Jonagold’. However, these genotypic differences were statistically not significant. The application of the lower levels of selenium in 2018 resulted in significantly lower selenium contents for both varieties. Again, the influence of the variety on the selenium content was not significant. The results of the present study are in line with published data. A significant increase of the selenium content resulting from biofortification with foliar fertilization has already been observed by other research groups in a variety of plant foods, especially on vegetables [16,17,18,19,21,22,25]. In those studies, the dosage form and the fertilizer level played a significant role. It was found that selenate leads to higher selenium accumulations than selenite [16,17,18,19] and the selenium content in the plants increased with increasing application level [16,18,19,20,21]. In the present experiments, no significant difference was found between the two forms of the selenium that were applied.

An increase in selenium concentration was also observed in different fruits. Pezzarossa et al. carried out a biofortification with 1.0 mg Se/L in the form of sodium selenate on peach (*Prunus persica* Batch. cv. Flavorcrest) and pear (*Pyrus communis* L. cv. ‘Conference’) and increased the selenium concentration in the fruits from < 0.1 µg Se/100 g f.w. to 0.9 µg Se/100 g f.w. and 3.6 µg Se/100 g f.w., respectively [26]. With regard to phytotoxicity resulting from fertilization with selenium, only slight damages on the fruits were observed in the year 2017. With reduced selenium levels in the follow-up year, there was no damage, anymore. However, moderate damages on the leaves were present in both years.

The use of selenium in a long-term cultivation program already showed good experience in Finland. Here, selenium fertilization has been carried out for many years on a national and compulsory basis. No corresponding ecological problems have been identified [39,40].

### 3.2. PPO Activity

PPO are very important enzymes, especially in apples, as quick browning of freshly cut apples is not accepted by the consumer [41]. Further, formation of the brown colored melanins has not yet been investigated with regard health risks. Usually, PPO substrates, small phenolic compounds, are still regarded being more health-beneficial [42,43,44,45].

**Figure 1:**
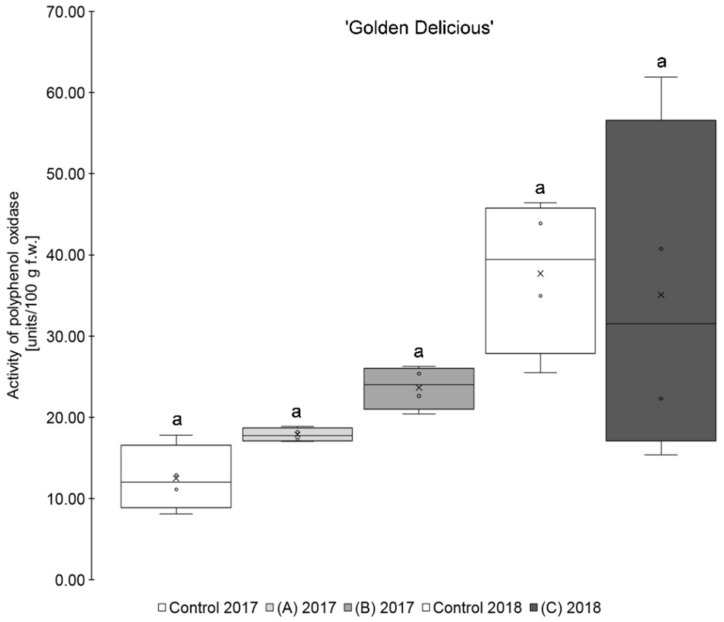
Polyphenol oxidase activity in units/100 g fresh weight (f.w.) for the apple samples of the variety ‘Golden Delicious’. Foliar Se application per hectare and meter canopy height: 0.15 kg as selenite (A), or selenate (B), 0.075 kg as selenate (C) (*n* = 4). Different letters are significantly different (*p* < 0.05).

**Figure 2:**
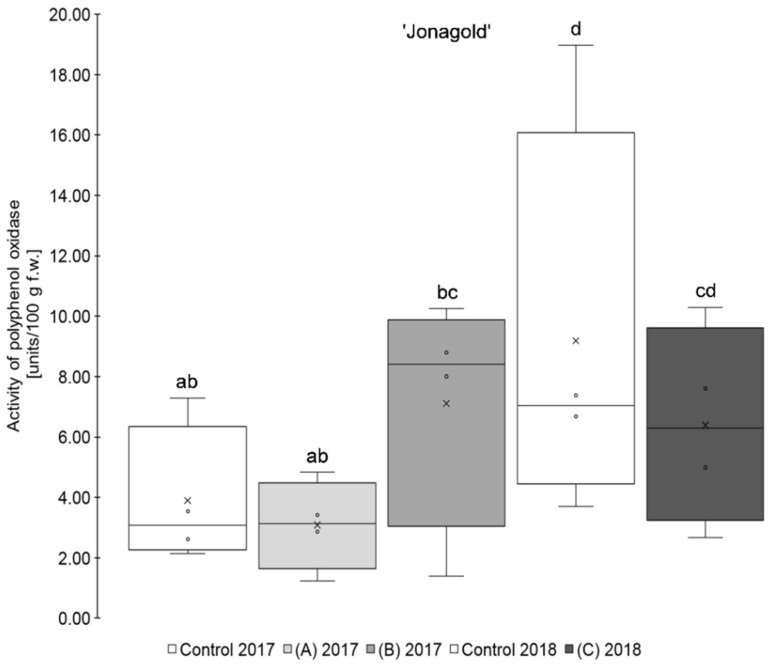
Results of polyphenol oxidase activity in units/100 g fresh weight for the apple samples of the variety ‘Jonagold’. Foliar Se application per hectare and meter canopy height: 0.15 kg as selenite (A), or selenate (B), 0.075 kg as selenate (C) (*n* = 4). Different letters are significantly different (*p* < 0.05).

The results show that the application of a higher amount of selenium (0.15 kg Se/ha x m CH), regardless of the form of selenium used, led, on average, to a higher PPO activity than those of the untreated controls for the two varieties ‘Golden Delicious’ and ‘Jonagold’ (Table 1, Figure 1; Figure 2). When on the other hand, the amount of selenium applied was lower (0.075 kg Se/ha x m CH), a lower PPO activity when compared to the controls was observed, being also valid for both cultivars. However, these differences between biofortified apples and the corresponding controls were not statistically significant. Smoleń et al. found increased PPO activities in comparison to the untreated controls, when performing a biofortification of potatoes (*Solanum tuberosum* L., cv. ‘Vineta’) with selenium (6.3 µM in the form of sodium selenite) and iodine, with being also not significant [46].

Furthermore, it was observed that the standard deviation of the measurements was - except for ‘Golden Delicious’ in 2018 - lower in the Se treatments as compared to the corresponding controls. The coefficient of variation ranged between 25.2 and 73.1% (mean 47.7%) in the controls, whereas, in the selenium biofortified apples, the values were between 4.8 and 59.4% (mean 38.5%). Holderbaum et al. also observed high variation coefficients of PPO in four apple cultivars at initial, intermediary, and final fruit development stages [47]. Reinkensmeier et al. measured low variation coefficients in selected varieties (e.g., ‘Golden Delicious’ and ‘Jonagold’) [48]. Smoleń et al. also found a high variation in PPO activity in potatoes as compared to the control with regard to the influence of a biofortification with selenium [46].

In addition, variety-specific differences in the PPO activity were observed. Significantly higher PPO activity was measured for ‘Golden Delicious’ as compared to ‘Jonagold’ in 2018 and partly also in 2017. Thus, ‘Golden Delicious’´s PPO activity was in a range between 12.50 and 37.69 units/100 g fw, whereas ‘Jonagold’s’ PPO activity was significantly lower and between 3.09 and 9.19 units/100 g f.w. Variety-specific differences of PPO activity were also observed by Holderbaum et al. [47], Kolodziejczyk et al. [31], and Kschonsek et al. [49]. The latter investigated various apple varieties, including ‘Golden Delicious’, which had the highest PPO activity of all the tested varieties [49]. This is in line with the results that were obtained here.

In addition to the variety influence, there were also differences in the PPO activity between both growth seasons, which can be explained by an influence of the different ecophysiological conditions of the crop years, like the sunshine duration and the resulting UV radiation [50]. This difference was significant for ‘Golden Delicious’, but not for ‘Jonagold’. Kolodziejczyk et al. have already observed differences in PPO activity within one variety in two consecutive years on a number of different apple varieties harvested in 2007 and 2008 [31]. Other research groups already investigated the influence of a UV-C treatment, which is an important postharvest treatment and influence on the PPO activity. For example, Manzocco et al. observed an inactivation of PPO and the prevention of enzymatic browning in ‘Golden Delicious’ apples by UV-C radiation [51]. Müller et al. also found a reduction of PPO activity in apple juices, when apples have been treated with UV-C light. In contrast, treatment with UV-B radiation did not show any effects [52]. Additionally, reduced PPO activities after UV-C treatments were observed in other vegetable crops [53,54].

### 3.3. Total Phenolic Content (TPC)

**Figure 3:**
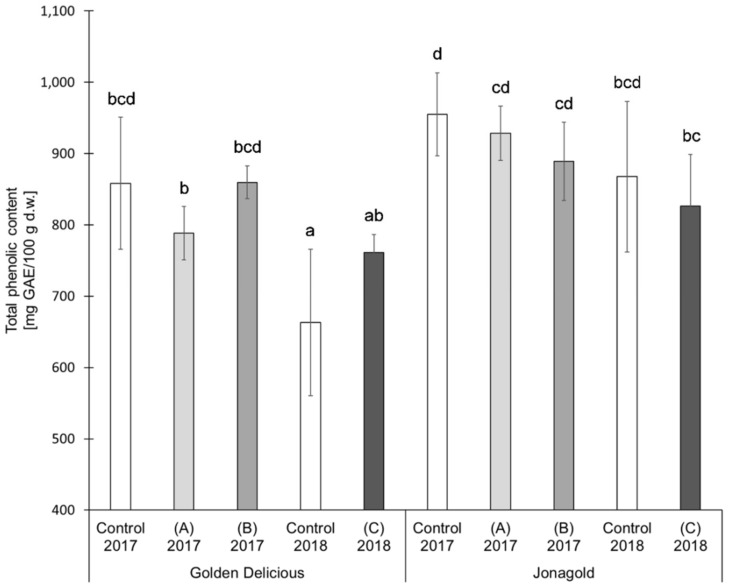
Total phenolic content (TPC) in mg GAE/100 g d.w. for the apple samples, depending on harvest year, apple variety, and form of selenium. Foliar Se application per hectare and meter canopy height: 0.15 kg as selenite (A), or selenate (B), 0.075 kg as selenate (C) (*n* = 4). Different letters are significantly different (*p* < 0.05).

The results of the TPC determination showed the following trends for the two varieties ‘Golden Delicious’ and ‘Jonagold’ (Table 1 and Figure 3). The application of selenite led on average to lower TPC values when compared to the untreated controls. The tendencies in the application of selenate were different, depending on the amount of fertilizer. Higher levels led to lower or constant TPC values, whereas lower levels of selenate tended to higher TPC values as compared to the untreated controls. However, the differences resulting from a biofortification with selenium were not statistically significant.

An increasing TPC of selenium biofortified produce has already been found by other research groups: Bachiega et al. performed an application of 50 µM selenate to broccoli, which led to a significant increase in TPC [25]. In onion (*Allium cepa* L., cv. ‘Hercules’), Põldma et al. observed that an application of 50 µg/mL selenate via foliar treatment led to increased TPC when compared to the untreated controls, whereas a higher level with 100 µg/mL resulted in lower TPC [55]. In tomatoes, Schiavon et al. found that selenate in low concentrations also led to an increase in TPC, when performing foliar fertilization of up to 20 mg Se/plant [21]. In a follow-up study on radish in 2016, an increase in TPC of 10% in the leaves when compared to the controls was recorded [22]. Hawrylak-Nowak found that the application of a moderate level of selenite (63.3 µM) applied via foliar fertilization led to enhanced TPC with a maximum increase of 43.9% in basil leaves *(Ocimum basilicum* L.) [18].

TPC also shows variety-specific differences between ‘Golden Delicious’ and ‘Jonagold’, with ‘Jonagold’ having higher values. The mean values of the controls were 858.4 mg GAE/100 g d.w. for the season 2017 and 663.0 mg GAE/100 g d.w. for the season 2018 for ‘Golden Delicious’, while the values for ‘Jonagold’ were 954.7 mg GAE/100 g d.w. and 867.7 mg GAE/100 g d.w., respectively. Variety-specific differences in TPC have already been described by other research groups. In the studies that were described by Kschonsek et al. and Xu et al., the TPC varied in the different apple varieties [49,56,57]. In 2018, Kschonsek et al. measured the TPC in 15 different apple cultivars and studied the peel and the fruit flesh, separately. In the peel, TPC was in a range between 521.9 mg GAE/100 g and 1590.5 mg GAE/100 g d.w. ‘Golden Delicious’ had the lowest TPC with a content of 521.9 mg GAE/100 g d.w., whereas the TPC of ‘Jonagold’ was 1224.2 mg GAE/100 g d.w. The amounts in the flesh were 136.5 mg GAE/100 g d.w. for ‘Golden Delicious’ and 177.5 mg GAE/100 g d.w. for ‘Jonagold’ [49]. When comparing the results of the selenium biofortified apples of the present study with those that were obtained by Kschonsek et al. [49], the TPC values of ‘Golden Delicious’ were in a comparable range, whereas the TPC of ‘Jonagold’ apples was somehow much lower in the present study.

Besides a genotypic influence, it is also obvious that seasonal influences that result from a differing ecophysiology might lead to differences. In 2018, lower TPC were measured for both varieties when compared to the previous season. For the summer 2018, a sunshine duration of 756 h, an average rainfall of 88.5 L/m², and an average temperature of 19.8 °C were determined in the area of Osnabrück, Germany, where apples were grown. In comparison, the sunshine duration of the previous year 2017 was only 549.4 h with an average rainfall of 224.3 L/m² and an average temperature of 18.2 °C [50]. The difference in the amount of rain only plays a marginal role because of the use of artificial irrigation. Consequently, sunshine duration in particular seems to be the dominant influence on the level of the TPC. Moreover, it is not really the sunshine duration, but its direct correlation to UV radiation. Eichholz et al showed that light intensity and quality are some of the most effective factors on the biosynthesis of phenolic compounds in white asparagus (*Asparagus officinnalis* L., cv. ‘Gijnlim’) on the basis of UV-B treatments [58]. The influence of UV-B has been recently reviewed by Neugart and Schreiner [59]. Scattino et al. could also demonstrate that a postharvest UV-B irradiation induced changes of TPC in peaches (*Prunus persica* L., cv. ‘Suncrest’) and nectarines (*Prunus persica* var. *nucipersica*, cv. ‘Big Top’) [60]. A higher TPC was expected for the apples in comparison to the previous year based on these results and the present results of year 2018, in which UV-B radiation was more intensive. However, the TPC of samples from 2017 was higher (Table 1 and Figure 3).

There is an inverse correlation between PPO activity and TPC, because the enzyme catalyzes the oxidation reaction of phenolic compounds to quinones, which further react to brown colored polymeric melanins [31,42]. This could explain the influence of the selenium biofortification as well as the variety-specific differences, where lower PPO activities are associated with higher TPC and higher enzyme activities with lower TPC.

At an application level of 0.15 kg Se/ha (selenite or selenate), higher PPO activities and lower TPC were measured as compared to the untreated control. PPO activity and TPC showed that biofortification at an application level of 0.075 kg Se/ha in the form of sodium selenate—when compared to the untreated controls—resulted in significant lower enzyme activities on the one hand and a significant increase of the TPC on the other hand. With regard to genotype, ‘Jonagold’ showed significantly lower enzyme activity than ‘Golden Delicious’, which resulted in a lower degradation of phenolic compounds.

An increased PPO activity is undesirable, because the enzymatically induced reaction of phenolic compounds leads to a degradation of the phenolic compounds and, thus, reduces the nutritional value of apples and apple products and has a negative influence on sensory properties. The consumer does not accept fast brown of apples and polymeric polyphenolic melanins might contribute to a certain astringency of food products [58]. Smaller phenolic compounds have positive effects on human health, due to their antioxidant, anti-inflammatory, and antimicrobial properties [42,43,44,45]. However, high PPO activities in the context of allergenicity of different apple varieties are negatively associated. Kschonsek et al. found that in some apple varieties high PPO activity are accompanied with lower concentrations of Mal d 1 [49]. This might be desirable in order to provide consumers with apple varieties of low allergenic potential.

There are further different data available in the literature regarding the correlation of PPO activity and TPC. Song et al. found positive correlations between PPO and TPC based on studies of ten apple varieties [61]. In contrast, Kolodziejczyk et al. found no correlation between these two parameters on the basis of 22 apple varieties [31]. Allahveran et al. performed biofortification with ascorbic acid and citric acid on apples of the variety ‘Red Spur’ and determined the PPO activity and TPC among other parameters. There, biofortification led to a significant increase in TPC and a decrease in PPO activity [62].

### 3.4. Antioxidant Activity (AOA)

The AOA of the apple samples was determined while using the two well-known assays TEAC and ORAC, which are based on different reaction mechanisms and, thus, different evaluations of AOA can be done. The ORAC assay bases on hydrogen transfer and measures the antioxidant inhibition being induced by peroxyl radicals. It represents a biologically relevant mechanism and the antioxidant activity is determined over time, so that the potential effects of secondary antioxidant compounds can also be measured and an underestimation can be prevented. The TEAC assay is easy to perform. Therefore, it is often used and there are many comparative values in the literature. Its mechanism bases on electron transfer reactions. It is comparatively insensitive to pH and determines both hydrophilic and lipophilic antioxidants. The TEAC is well suited for the determination of antioxidant activity in phenolic rich samples, such as apples, as the ABTS^•+^ reacts quickly with antioxidants and many phenolic compounds of low redox potential [63].

**Figure 4:**
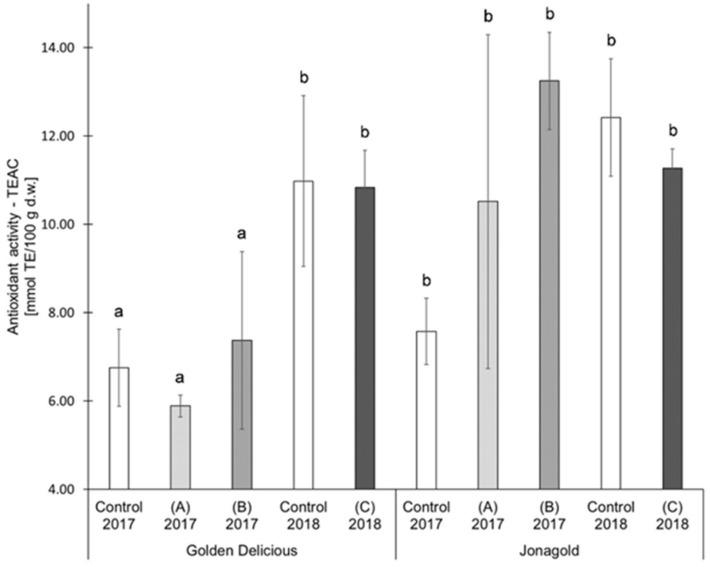
Results of the antioxidant activity (Trolox Equivalent Antioxidant Capacity Assay (TEAC) assay) in mmol TE/100 g d.w. for the apple samples. Foliar Se application per hectare and meter canopy height: 0.15 kg as selenite (A), or selenate (B), 0.075 kg as selenate (C) (*n* = 4). Different letters are significantly different (*p* < 0.05).

**Figure 5:**
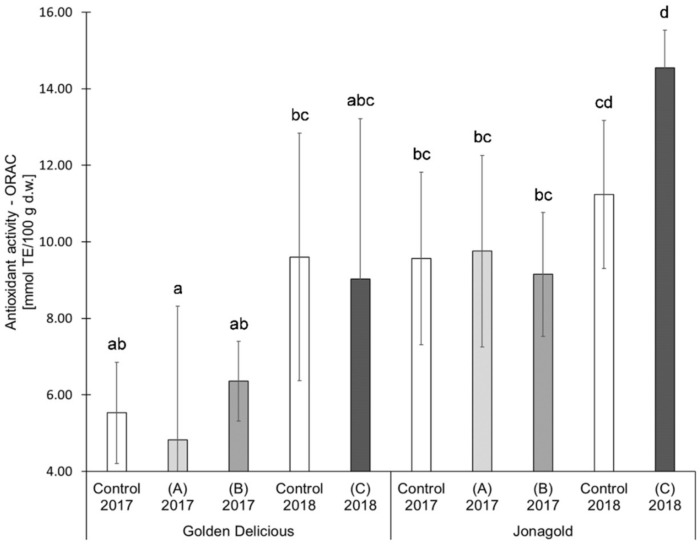
Results of the antioxidant activity (Oxygen Radical Absorbance Capacity Assays (ORAC) assay) in mmol TE/100 g d.w. for the apple samples. Foliar Se application per hectare and meter canopy height: 0.15 kg as selenite (A), or selenate (B), 0.075 kg as selenate (C) (*n* = 4). Different letters are significantly different (p < 0.05).

In the present study, selenium biofortification did not reveal any clear tendencies of an influence on AOA. The two studied varieties were differently affected (Table 1, Figure 4, Figure 5): ‘Jonagold’ mostly showed a higher AOA (measured with TEAC) resulting from the biofortification and independently of the selenium form and the level of application, whereas the AOA of ‘Golden Delicious’ was only slightly influenced by the biofortification. The increase in AOA was significant for ‘Jonagold’ in 2017 with the application of 0.15 kg Se/ha in the form of selenite and in the form of selenate. Related to the dose of selenium, the evaluation of the AOA with the TEAC assay provided the following results: The application of 0.15 kg selenate in the season 2017 led to higher AOA in both varieties. The treatment with 0.15 kg selenite resulted in lower AOA for ‘Golden Delicious’ and higher AOA for ‘Jonagold’ compared to the corresponding controls (Table 1 and Figure 4). When 0.075 kg Se/ha in the form of selenate were applied in 2018, a slight, non-significant reduction in AOA was observed for both varieties.

For ‘Golden Delicious, the determination of the AOA with the ORAC assay showed the same tendencies as the TEAC values. However, differences were found for ‘Jonagold’: While the TEAC value for an application level of 0.15 kg selenate was significantly higher compared to the control, the ORAC value was lower when compared to the control. The treatment with 0.075 kg selenate led to an increase of the ORAC value in comparison to the decrease of the TEAC value.

An increase in AOA due to biofortification with selenium has also been noted by other researchers: Ríos et al. were able to show that increasing doses of selenite and selenate lead to an increase in AOA, as measured by FRAP and DPPH assay in lettuce plants (*Lactuca sativa* L. cv ‘Philipus’). Selenate showed higher AOA when compared to selenite [19]. Põldma et al. determined an increase in AOA, as measured by TEAC, in onions (*Allium cepa* L. cv. ‘Hercules’) at a dose of 50 µg/mL Se [55]. Bachiega et al. found a significant increase in AOA as a result of a biofortifiaction of broccoli in addition to a significantly higher TPC. There, 50 µM selenate was used as fertilizer. The positive correlation can be explained by the fact that phenolic compounds represent the largest group of antioxidant active substances in broccoli [25]. Additionally, Ekanayake et al. observed an increase of the AOA of lentils (*Lens culinaris* cv. ‘Medikus’), due to a biofortification with selenium [17].

Variety-specific differences occurred in the present study. AOA of ‘Jonagold’ was higher in both years of cultivation than for ‘Golden Delicious’. Variety-specific differences in the AOA of apples have already been described in the literature by Xu et al., Kschonsek et al., and Wojdylo et al. [56,57,64]. In those studies, the higher AOA of the variety ‘Jonagold’ as compared to ‘Golden Delicious’ were found. Kschonsek et al. determined the AOA in the skin and the flesh of different apple varieties: TEAC values of 2.4 mmol TE/100 g d.w. for ‘Golden Delicious’ and 9.1 mmol TE/100 g d.w. for ‘Jonagold’ were measured for the peel, ORAC values were 8.6 mmol TE/100 g d.w. and 24.6 mmol TE/100 g d.w., respectively [56], and are therefore comparable with the results that were obtained in the present study. Wojdylo et al. determined the AOA using the TEAC assay and measured an AOA of 88.6 ± 6.7 mmol TE/100 g d.w. for ‘Golden Delicious’ and 181.9 ± 0.9 mmol TE/100 g d.w. for ‘Jonagold’ [64].

Xu et al., Kschonsek et al., and Wojdylo et al. were able to show that there are significant positive correlations between TPC (measured according to Folin-Ciocalteu or via HPLC) and AOA [56,57,64]. Kschonsek et al. and Wojdylo et al. also determined that the TPC was different, both between the individual substance groups of the polyphenols and between the individual compounds [56,64]. There, Kschonsek et al measured the highest positive correlation between flavanols and ORAC. Those compounds are the major contributors to AOA. Within the flavanols, epicatechin had the strongest influence on the intensity of the AOA [56]. Wojdylo et al. found the highest correlations between AOA and procyanidins and hydroxycinnamic acids, while using the TEAC, FRAP, and DPPH assay. The different AOA of the individual varieties are, therefore, due to the different composition of the phenolic compounds, as these show different antioxidant capacities and potentials [64].

An influence of the weather can also be deduced when comparing the controls from the years 2017 and 2018, similarly to the TPC values. AOA measured as TEAC of the untreated ‘Golden Delicious’ apples from the season 2017 was 6.76 mmol TE/100 g d.w. In the following year, an increase of 62.4% was observed with a value of 10.98 mmol TE/100 g d.w. For ‘Jonagold’, AOA of 7.58 mmol TE/100 g d.w., and 12.42 mmol TE/100 g d.w. were measured, corresponding to an increase of 63.9%. With regard to ORAC values, an increase of the AOA could also be observed for ‘Golden Delicious’, being 73.6% (5.53 mmol TE/100 g d.w. in 2017 and 9.60 mmol TE/100 g d.w. in 2018). For ‘Jonagold’, on the other hand, only a moderate increase of 17.6% was observed (9.56 mmol TE/100 g d.w. in 2017 and 11.24 mmol TE/100 g d.w. in 2018).

### 3.5. Qualitative and Quantitative Determination of Phenolic Compounds Using HPLC-MS^n^

The following major phenolic compounds could be identified and quantitatively determined in the apple samples of the varieties ‘Golden Delicious’ and ‘Jonagold’ while using HPLC-MS^n^ analysis: the dihydrochalcones phloretin-2-xylosyl-glucoside and phloretin-2-glucoside, the flavan-3-ol epicatechin, and a procyanidin dimer, and a procyanidin trimer, the hydrocinnamic acid derivatives caffeoyl glucoside and chlorogenic acid, as well as the flavonols quercetin-3-*O*-galactoside, quercetin-3-*O*-xyloside and quercetin-3-*O*-glucoside. Chlorogenic acid, epicatechin, caffeoyl glucoside, and the procyanidin trimer were the main compounds in the samples.

**Figure 6:**
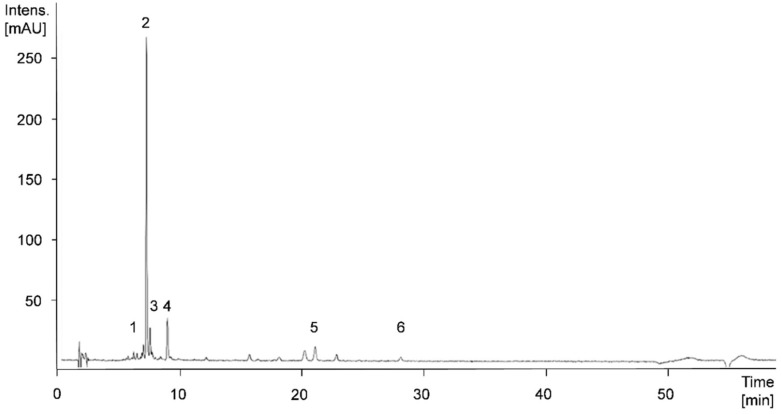
High Performance Liquid Chromatography Mass Spectrometry (HPLC-MS)^n^-chromatogram at 280 nm of apple cultivar ‘Jonagold’, biofortified with 0.075 kg Se/ha in the form of selenate in 2018. Peak numbers: 1, procyanidin dimer; 2, chlorogenic acid; 3, epicatechin; 4, procyanidin trimer; 5, phloretin-2-xylosyl-glucoside; 6, phloretin-2-glucoside.

Figure 6 shows an exemplary HPLC-chromatogram at 280 nm of an apple sample of the cultivar ‘Jonagold’, biofortified with 0.075 kg Se/ha in the form of selenate, produced in the year 2018. In Table 2, the total phenolic content as the sum of all individual phenolic compounds, the content of the four main phenolic compounds and their respective shares of the total phenolic content of the biofortified apples of the varieties ‘Golden Delicious’ and ‘Jonagold’ with the various selenium applications, as well as the corresponding untreated controls of the years 2017 and 2018 are listed. For ‘Golden Delicious’, no data were available from the season 2018.

In the apple varieties ‘Golden Delicious’ and ‘Jonagold’ hydroxycinnamic acid derivatives could be identified, in particular. The main phenolic compound was chlorogenic acid, with shares between 22.3% and 31.6% of the total sum of individual phenolic compounds. Furthermore, the apples were rich in epicatechin, caffeoyl glucoside, and a procyanidin trimer. Based on tentative structure elucidation in the present study and literature descriptions, this trimer is suggested being procyanidin C1 [65].

In a recent review, Rana and Bhushan compiled and evaluated a large number of data of the analysis of phenolic compounds in apples [66]. It was found that the phenolic compounds of the subclasses flavonols, dihydrochalcones, flavan-3-ols, and phenolic acids have already been identified in apples of various varieties. Epicatechin, procyanidin B2, chlorogenic acid, phloridzin, caffeic acid, and quercetin derivatives are the major components. Dhyani et al. and Zardo et al. identified chlorogenic acid and epicatechin as major components in ‘Golden Delicious’ [67,68]. In 2005 and 2006, Wojdylo et al. determined the phenolic compounds in 69 apple cultivars, including ‘Golden Delicious’ and ‘Jonagold’. In both varieties, most of all oligomeric procyanidins, and chlorogenic acid were found, whereas ‘Jonagold’ contained more epicatechin and chlorogenic acid in comparison [64]. Deviating results were obtained in the study that was described by Kschonsek et al. In those apple samples from the varieties ‘Golden Delicious’ and ‘Jonagold’, mainly flavonols were determined, whereas quercetin derivatives, especially hyperosides, were the main components. Only 5.3% of chlorogenic acid (‘Golden Delicious’) and 1.6% (‘Jonagold’) were present in the peel. The flesh only contained very small amounts of phenolic compounds [56].

In the present study, biofortification with selenium showed different effects for ‘Golden Delicious’ and ‘Jonagold’ with regard to the content and proportion of the individual phenolic compounds. In particular, the four phenolic compounds that are listed in Table 2 have been influenced resulting from the biofortification. Further phenolic compounds were not significantly affected.

The phenolic profile of ‘Golden Delicious’ was comparatively insensitive, whereas, in the case of ‘Jonagold’, the application of selenate, in particular, led to changes in the proportions of the individual phenolic compounds. Here, significant differences in the content of the procyanidin trimer and caffeoyl glucoside occurred in the samples from 2017, when the higher amount of selenate was applied.

Lower total contents of individual phenolic compounds were measured when compared to the corresponding controls in all selenium applications on the ‘Jonagold’ variety. These results correspond to the TPC results. The application of 0.15 kg selenate/ha in 2017 resulted in a significantly lower concentration and proportion of the procyanidin trimer in ‘Jonagold’ when compared to the untreated control, whereas the concentration and proportion of caffeoyl glucoside significantly increased from 3.4% to 9.2%. AOA measured with the TEAC assay was highest in the apples of the selenate applications, which suggests that, due to the high proportion, caffeoyl glucoside is mainly responsible for AOA in ‘Jonagold’. This trend - the increase of caffeoyl glucoside and the TEAC-value - was also observed at the biofortification of 0.15 kg selenite in 2017, but to a lower extent compared to the selenate treatments.

The application of the lower level of selenate (0.075 kg/ha) in 2018 did not confirm the observations from the previous year, as no significant changes between control and selenium-biofortified apple samples have been observed. This observation might be related to the lower amount of selenium applied. With regard to the AOA, very similar values were also measured in the control and biofortified samples. Based on the results of the ‘Jonagold’ samples from 2017, correlations between the individual phenolic compounds and their AOA can be concluded. With a high content of the procyanidin trimer, low AOA with TEAC and high AOA with ORAC were measured. On the other hand, high concentrations of caffeoyl glucoside were associated with high AOA by TEAC and low AOA by ORAC. These results further suggest that these two phenolic compounds have different AOA and - due to the different reaction mechanisms of both antioxidant assays - the AOA of different phenolic compounds were determined and secondary antioxidant products were additionally measured when using the ORAC assay [69]. The individual contribution of the phenolic compounds should be analyzed by HPLC-online TEAC because of the different antioxidant capacities and potentials of the phenolic compounds.

Various research groups have already observed a change in the phenolic profile that results from a biofortification with selenium: D’Amato et al. found an increase of oleacein, ligustroside aglycone, and oleocanthal in olive oil, whose contents increased by 32% to 57% compared to the untreated control [24]. In a follow-up study on rice in 2018, hydroxybenzoic acids and hydroxycinnamic acids were identified, with an increase in ferulic acid and salicylic acid, whereas the concentrations of gallic acid decreased [16]. Schiavon et al. carried out experiments with the biofortification of radish leaves and roots. In roots, the antioxidant flavonoids naringenin chalcone and kaempferol showed enhanced concentrations and a decrease of cinnamic acid derivatives was observed. In leaves, the hydroxycinnamic acids, especially kaempferol derivatives, were increased, caffeic acid did not increase, and other identified phenolic compounds did not show any variation in concentration or decreased [21]. Pezzarossa et al. performed an application of 1 mg Se/L (as sodium selenate) in tomatoes (*Solanum lycopersion* cv. ‘Red Bunch’), in which a significant increase of quercetin was observed in addition to a decrease of β-carotene and lycopene. Rutin was not influenced [70].

## 4. Conclusions

The aim of the present study was to investigate the biofortification of apples with selenium and its influence on the selenium content, phenolic compounds, and the properties associated with these substances.

The selenium content, PPO activity, TPC, AOA, and the composition of the phenolic compounds were influenced in different ways, depending on the conditions of biofortification. Here, the level of application and the form of selenium used played a major role. Furthermore, variety-specific differences in the level of the parameters could be identified. ‘Golden Delicious’ and ‘Jonagold’ behaved differently in some cases. The influence of ecophysiological conditions, especially the different sunshine duration, was also identified.

Biofortification led to a significant increase in the selenium content in the apples. Here, the level of selenium accumulation in the fruits mainly depended on the level of fertilizer. The form of selenium used only played a minor role.

When increasing the selenium content in the apples, the selenium supply with meeting the nutritional recommendations, can be improved. An apple of the variety ‘Golden Delicious’ with an average weight of 220 g (± 16,5 g) in 2017 and 213 g (± 15,0 g) in 2018 can, therefore, cover the daily requirement by approximately 17–20%. Taking into account the higher average weight of ‘Jonagold’ with values of 273 g (± 31,5 g) in 2017 and 255 g (± 25,3 g) in 2018, the consumption of one apple can cover 20–25% of the daily requirement of selenium.

Further, it can be stated that selenium biofortification has a stabilizing effect on the activity of PPO, as the values between the apples varied less. The PPO activity was also related to the amount of selenium fertilizer used—higher levels led to increased activities. This can also explain the TPC, as higher selenium levels resulted in constant or lower values, because more phenolic compounds are potentially degraded. A stabilized PPO activity will enable stable browning reactions when focusing on processed apple products in the future. A quick browning of freshly cut apples is not accepted by the consumer. Further, the formation of the brown colored melanins has not yet been investigated with regard health risks. Usually, PPO substrates—small phenolic compounds—are still regarded being health-beneficial principles.

In further studies, HPLC-onlineTEAC coupling should be used to investigate the AOA of individual phenolic compounds, as the results that were obtained by HPLC-DAD-ESI-MS^n^ indicate a certain variability of the phenolic compounds, providing different AOA. Based on the results, it can be concluded for the present study that the phenolic compounds contained, especially the procyanidin trimer (suggested to be procyanidin C1) and caffeoyl glucoside, have different AOA, which may also be different, depending on the variety. These should be further analyzed by HPLC-onlineTEAC. It has already been described in the literature that different phenolic compounds contribute differently to the total AOA. Riehle et al. determined the AOA of the individual phenolic compounds in *Cistus incanus* herbal tea infusions while using HPLC-onlineTEAC and found that the individual phenolic compounds had different AOA and different proportions of the total AOA of the samples [71]. Zietz et al. and Fiol et al. analyzed kale (*Brassica oleraceae* var. *Sabellica*) and found different AOA of the flavonoid glycosides and hydroxycinnamic acid derivatives contained in the samples [72,73].

When the phenolic profiles with their corresponding single antioxidant capacities are evaluated, it is possible to conclude also for their bioavailability and even bioactivity of the polyphenols.

## Figures and Tables

**Table 1:** Results of the determination of the selenium content, polyphenol oxidase activity, total phenolic content, and antioxidant activity in all apple samples. Data are given as mean value ± standard deviation (*n* = 4). In each column, means followed by different letters are significantly different (*p* < 0.05).

Variety and Year of Cultivation	Application *	Se [µg/100 g f.w.]	PPO [units/100 g f.w.]	Total Phenolic Compound [mg GAE/100 g d.w.]	TEAC [mmol TE/100 g d.w.]	ORAC [mmol TE/100 g d.w.]
Golden Delicious 2017	control	0.4 ± 0.2 ^a^	12.50 ± 4.05 ^,b^	858.4 ± 92.5 ^b,c,d^	6.76 ± 0.87 ^a^	5.53 ± 1.32 ^a,b^
	0.15 kg selenite	5.6 ± 0.6 ^b^	17.85 ± 0.85 ^ab^	788.3 ± 37.3 ^b^	5.89 ± 0.25 ^a^	4.82 ± 3.50 ^a^
	0.15 kg selenate	5.6 ± 0.8 ^b^	23.67 ± 2.66^bc^	859.6 ± 23.0 ^b,c,d^	7.37 ± 2.01 ^a^	6.36 ± 1.04 ^a,b^
Golden Delicious 2018	control	< 0.2 ^a^	37.69 ± 9.50 ^d^	663.0 ± 102.8 ^a^	10.98 ± 1.93 ^b^	9.60 ± 3.23 ^bc^
	0.075 kg selenate	2.7 ± 0.8 ^c^	35.07 ± 20.84 ^c,d^	761.3 ± 25.1 ^a,b^	10.83 ± 0.84 ^b^	9.03 ± 4.19 ^a,b,c^
‘Jonagold’ 2017	control	0.4 ± 0.2 ^a^	3.90 ± 2.34 ^a^	954.7 ± 58.1 ^d^	7.58 ± 0.75 ^a^	9.56 ± 2.26 ^b,c^
	0.15 kg selenite	5.6 ± 1.1 ^b^	3.09 ± 1.50 ^a^	928.4 ± 37.9 ^c,d^	10.51 ± 3.78 ^b^	9.76 ± 2.51 ^b,c^
	0.15 kg selenate	4.5 ± 1.6 ^b^	7.11 ± 3.92 ^a^	889.1 ± 54.6 ^c,d^	13.24 ± 1.10 ^b^	9.15 ± 1.62 ^b,c^
‘Jonagold’ 2018	control	< 0.2 ^a^	9.19 ± 6.72 ^a^	867.7 ± 105.5 ^b,c,d^	12.42 ± 1.33 ^b^	11.24 ± 1.93 ^c,d^
	0.075 kg selenate	2.0 ± 0.3 ^c^	6.39 ± 3.29 ^a^	826.5 ± 72.1 ^b,c^	11.27 ± 0.44 ^b^	14.55 ± 0.98 ^d^

* Foliar spray rate per hectare and meter canopy height.

**Table 2:** Results of the determination of phenolic compounds using HPLC-MS^n^. Data are in average ± standard deviation. The total phenolic content in mg/100 g d.w. was calculated by the sum of all quantitative determined phenolic compounds. For the four main phenolic compounds the content in mg/100 g d.w. and the percentage share is given. In each column, means followed by different letters are significantly different (*p* < 0.05).

Variety and Year of Cultivation	Application *	Σ [mg/100 g d.w.]	Chlorogenic Acid [mg/100 g d.w.] %		Epicatechin [mg/100 g d.w.] %		Procyanidin Trimer [mg/100 g d.w.] %		Caffeoyl glucoside [mg/100 g d.w.] %	
Golden Delicious’ 2017	control	126.86 ± 14.04 ^a,b^	32.73 ± 2.92 ^a^	25.9	13.05 ± 3.82 ^a,b^	10.2	9.78 ± 2.30 ^a^	7.6	14.07 ± 1.03 ^d^	11.2
	0.15 kg selenite	119.73 ± 42.13 ^a^	33.62 ± 3.24 ^a^	31.6	12.15 ± 4.75 ^a,b^	10.0	9.07 ± 3.46 ^a^	7.5	14.27 ± 0.63 ^d^	13.8
	0.15 kg selenate	123.66 ± 12.96 ^a,b^	33.49 ± 2.22 ^a^	28.4	13.18 ± 1.64 ^a,b^	10.7	8.33 ± 1.37 ^a^	6.7	13.90 ± 1.72 ^c,d^	11.3
Golden Delicious’ 2018	control	-	-	-	-	-	-	-	-	-
	0.075 kg selenate	-	-	-	-	-	-	-	-	-
‘Jonagold’ 2017	control	152.52 ± 27.88 ^b^	36.42 ± 13.30 ^a^	24.0	19.50 ± 9.81 ^b^	12.2	18.61 ± 7.48 ^b^	11.8	5.11 ± 0.17 ^a^	3.4
	0.15 kg selenite	130.88 ± 4.38 ^a,b^	29.30 ± 4.22 ^a^	22.3	17.11 ± 2.87 ^a,b^	13.1	14.74 ± 4.11 ^a,b^	11.3	7.68 ± 1.46 ^a^	5.9
	0.15 kg selenate	124.74 ± 11.94 ^a,b^	29.41 ± 2.82 ^a^	23.5	12.10 ± 1.40 ^a,b^	9.7	9.87 ± 1.87 ^a,^	7.9	11.23 ± 2.72 ^b,c,d^	9.2
‘Jonagold’ 2018	control	107.39 ± 8.22 ^a^	31.31 ± 0.91 ^a^	29.2	10.74 ± 1.66 ^a,b^	10.0	8.45 ± 0.32 ^a^	7.9	11.47 ± 1.20 ^b,c,d^	10.7
	0.075 kg selenate	104.15 ± 2.97 ^a^	27.07 ± 0.23 ^a^	26.0	9.60 ± 8.39 ^a^	9.1	8.09 ± 5.40 ^a^	7.7	11.28 ± 2.65 ^b,c,d^	10.8

* Foliar spray rate per hectare and meter canopy height.

## References

[1] Hirschi K.D. (2009). Nutrient biofortification of food crops. Annu. Rev. Nutr..

[2] Lawson P.G., Daum D., Czauderna R., Vorsatz C. (2016). Factors influencing the efficacy of iodine foliar sprays used for biofortifying butterhead lettuce (*Lactuca sativa*). J. Plant Nutr. Sci..

[3] Cakmak I. (2009). Agronomic Approaches in Biofortification of Food Crops with Micronutrients.

[4] Gupta M., Gupta S. (2017). An overview of selenium uptake, metabolism, and toxicity in plants. Front. Plant Sci..

[5] Poňavič M., Scheib A., Reimann C., Birke M., Demetriades A., Filzmoser P., O’Connor P. (2014). Distribution of selenium in European agricultural and grazing land soil. Chemistry of Europe’s Agricultural Soils.

[6] Winkel L.H., Vriens B., Jones G.D., Schneider L.S., Pilon-Smits E., Bañuelos G.S. (2015). Selenium cycling across soil-plant-atmosphere interfaces: A critical review. Nutrients.

[7] Alfthan G., Eurola M., Ekholm P., Venäläinen E., Root T., Korkalainen K., Hartikainen H., Salminen P., Hietaniemi V., Aspila P. (2015). Effects of nationwide addition of selenium to fertilizers on foods, and animal and human health in Finland: From deficiency to optimal selenium status of the population. J. Trace Elem. Med. Biol..

[8] Rayman M. (2012). Selenium and human health. Lancet.

[9] Kielliszek M. (2019). Selenium-fascinating microelement, properties and sources in food. Molecules.

[10] Kielliszek M., Błażejak S. (2016). Current knowledge on the importance of selenium in food for living organisms: A review. Molecules.

[11] Kipp A.P., Strohm D., Brigelius-Flohé R., Schomburg L., Bechthold A., Leschik-Bonnet E., Heseker H. (2015). German Nutrition Society (DGE) Revised reference values for selenium intake. J. Trace Elem. Med. Biol..

[12] Oster O., Prellwitz W. (1989). The daily dietary selenium intake of West German adults. Biol. Trace Elem. Res..

[13] Willers J., Heinemann M., Bitterlich N., Hahn A. (2015). Intake of minerals from food supplements in a German population—A nationwide survey. Food Nutr. Sci..

[14] Wortmann L., Enneking U., Daum D. (2018). German consumers’ attitude towards selenium-biofortified apples and acceptance of related nutrition and health claims. Nutrients.

[15] Statista Pro-Kopf-Konsum von Obst in Deutschland nach Art in den Jahren 2010/11 bis 2017/18 (in Kilogramm). https://de.statista.com/statistik/daten/studie/6300/umfrage/pro-kopf-verbrauch-von-obst-in-deutschland/.

[16] D’Amato R., Fontanella M.C., Falcinelli B., Beone G.M., Bravi E., Marconi O., Benincasa P., Businelli D. (2018). Selelium biofortification in rice (*Oryza sativa* L.) sprouting: Effects on Se yield and nutritional traits with focus on phenolic acid profile. J. Agric. Food Chem..

[17] Ekanayake L.J., Thavarajah D., Vial E., Schatz B., McGee R., Thavarajah P. (2015). Selenium fertilization on lentil (*Lens culinaris* Medikus) grain yield, seed delinium concentration, and antioxidant activity. Field Crop. Res..

[18] Hawrylak-Nowak B. (2008). Enhanced selenium content in sweet basil (*Ocimum basilicum* L.) by foliar fertilization. Veg. Crop. Res. Bull..

[19] Ríos J.J., Rosales M.A., Blasco B., Cervilla L.M., Romero L., Ruiz J.M. (2008). Biofortification of Se and induction of the antioxidant capacity in lettuce plants. Sci. Hort..

[20] Hawrylak-Nowak B. (2013). Comparative effects of selenite and selenate on growth and selenium accumulation in lettuce plants under hydroponic conditions. Plant Growth Regul..

[21] Schiavon M., dall’Acqua S., Mietto A., Pilon-Smits E.A.H., Sambo P., Masi A., Malagoli M. (2013). Selenium fertilization alters the chemical composition and antioxidant constituents of tomato (*Solanum lycopersicon* L.). J. Agric. Food Chem..

[22] Schiavon M., Berto C., Malagoli M., Trentin A., Sambo P., Dall’Acqua S., Pilon-Smits E.A.H. (2016). Selenium biofortification in radish enhances nutritional quality via accumulation of methyl-selenocystein and promotion of transcripts and metabolites related to glucosinolates, phenolics, and amino acids. Front. Plant Sci..

[23] Zhao Y., Wu P., Wang Y., Feng H. (2013). Different approaches for selenium biofortification of pear-jujube (*Zizyphus jujuba* M. cv. Lizao) and associated effects on fruit quality. J. Food Agric. Environ..

[24] D’Amato R., Proietti P., Onofri A., Regni L., Esposto S., Servili M., Businelli D., Selvaggini R. (2017). Biofortification (Se): Does it increase the content of phenolic compounds in virgin olive oil (VOO)?. PLoS ONE.

[25] Bachiega P., Salgado J.M., de Carvalho J.E., Ruiz A.L., Schwarz K., Tezotto T., Morzelle M.C. (2016). Antioxidant and antiproliferative activities in different maturation stages of broccoli (*Brassica oleracea* var. *italica*) biofortified with selenium. Food Chem..

[26] Pezzarossa B., Remorini D., Gentile M.L., Massai R. (2012). Effects of foliar and fruit addition of sodium selenate on selenium accumulation and fruit quality. J. Sci. Food Agric..

[27] Babalar M., Mohebbi S., Zamani Z., Askari M.A. (2019). Effect of foliar application with sodium selenate on selenium biofortification and fruit quality maintenance of ‘Starking Delicious’ apple during storage. J. Sci. Food Agric..

[28] Lawson P.G., Daum D., Czauderna R., Meuser H., Härtling J.W. (2015). Soil versus foliar iodine fertilization as a biofortification strategy for field-grown vegetables. Front. Plant Sci..

[29] (2014). DIN EN 13805: Foodstuffs—Determination of Trace Elements—Pressure Digestion, German Version DIN EN 13805:2014-12.

[30] (1994). DIN 38405-23: German standard methods for the examination of water, waste water and sludge—Anions (Group D)—Part 23: Determination of Selenium by Atomic Absorption Spectrometry (D 23).

[31] Kolodziejczyk K., Milala J., Sojka M., Kosmala M., Markowski J. (2010). Polyphenol oxidase activity in selected apple cultivars. J. Fruit Ornam. Plant Res..

[32] Gonzalez E.M., de Ancos B., Pilar Cano M. (1999). Partial characterization of polyphenol oxidase activity in raspberry fruits. J. Agric. Food Chem..

[33] Müller L., Gnoyke S., Popken A.M., Böhm V. (2010). Antioxidant capacity and related parameters of different fruit formulations. LWT Food Sci. Technol..

[34] Singleton V.L., Rossi J.A. (1965). Colorimetry of total phenolics with phosphomolybdic-phosphotungstic aid reagents. Am. J. Enol. Viticult..

[35] Re R., Pellegrini N., Proteggente A., Pannala A., Yang M., Rice-Evans C. (1999). Antioxidant activity applying an improved ABTS radical cation decolorization assay. Free Rad. Biol. Med..

[36] Ou B., Hampsch-Woodill M., Prior R.L. (2001). Development and validation of an improved oxygen radical absorbance capacity assay using fluorescein as the fluorescent probe. J. Agric. Food Chem..

[37] Neugart S., Baldermann S., Ngwene B., Wesonga J., Schreiner M. (2017). Indigenous leafy vegetables of Eastern Africa—A source of extraordinary secondary plant metabolites. Food Res. Int..

[38] Schmidt S., Zietz M., Schreiner M., Rohn S., Kroh L.W., Krumbein A. (2010). Identification of complex, naturally occurring flavonoid glycosides in kale (*Brassica oleracea* var. *sabellica*) by high-performance liquid chromatography diode-array detection/electrospray ionization multistage mass spectrometry. Rapid Commun. Mass Spectrom.

[39] Smoleń S., Kowalska I., Czernicka M., Halka M., Keska K., Sady W. (2016). Iodine and selenium biofortification with additional application of salicylic acid affects yield, selected molecular parameters and chemical composition of lettuce plants (*Lactuca sativa* L. var. *capitata*). Front. Plant Sci..

[40] Eurola M., Alfthan G., Aro A., Ekholm P., Hietaniemi V., Rainio H., Rankanen R., Venäläinen E. (2003). Results of the Finnish Selenium Monitoring Program 2000–2001.

[41] Nicolas J.J., Richard-Forget F.C., Goupy P.M., Amoit M., Aubert S.Y. (1994). Enzymatic browning reactions in apple and apple products. Crit. Rev. Food Sci. Nutr..

[42] Bravo L. (1998). Polyphenols: Chemistry, dietary sources, metabolism, and nutritional significance. Nutr. Rev..

[43] Kroon P., Williamson G. (2005). Polyphenols: Dietary components with established benefits to health?. J. Sci. Food Agric..

[44] Del Rio D., Costa L.G., Lean M.E.J., Crozier A. (2010). Polyphenols and health: What compounds are involved?. Nutr. Metab. Cardiovasc. Dis..

[45] Tomás-Barberán F.A., Andrés-Lacueva C. (2012). Polyphenols and health: Current state and progress. J. Agric. Food Chem..

[46] Smoleń S., Kowalska I., Skoczylas L., Liszka-Skoczylas M., Grzanka M., Halka M., Sady W. (2018). The effect of salicylic acid on biofortification with iodine and selenium and the quality of potato cultivated in the NFT system. Sci. Hortic..

[47] Holderbaum D.F., Kon T., Kudo T., Guerra M.P. (2010). Enzymatic browning, polyphenol oxidase activity, and polyphenols on four apple cultivars: Dynamics during fruit development. HortScience.

[48] Reinkensmeier A., Steinbrenner K., Homann T., Bußler S., Rohn S., Rawel H.M. (2016). Monitoring the apple polyphenol oxidase-modulated adduct formation of phenolic and amino compounds. Food Chem..

[49] Kschonsek J., Dietz A., Wiegand C., Hipler U., Böhm V. (2019). Allergenicity of apple allergen Mal d 1 as effected by polyphenols and polyphenol oxidase due to enzymatic browning. Lebens. Wiss. Technol..

[50] WetterKontor GmbH Monats-Und Jahreswerte Für Deutschland. https://www.wetterkontor.de/de/wetter/deutschland/monatswerte.asp?y=2018&m=15.

[51] Manzocco L., Quarta B., Dri A. (2009). Polyphenoloxidase inactivation by light exposure in model systems and apple derivatives. Innov. Food Sci. Emerg. Technol..

[52] Müller A., Noack L., Greiner R., Stahl M.R., Posten C. (2014). Effect of UV-C and UV-B treatment on polyphenol oxidase acitvity and shelf life of apple and grape juice. Innov. Food Sci. Emerg. Technol..

[53] Lei J., Li B., Zhang N., Yan R., Guan W., Brennan C.S., Gao H., Peng B. (2018). Effects of UV-C treatment on browning and the expression of polyphenol oxidase (PPO) genes in different tissues of *Agaricus bisporus* during cold storage. Postharvest Biol. Technol..

[54] Cirilli M., Caruso G., Gennai C., Urbani S., Frioni E., Ruzzi M., Servili M., Gucci R., Poerio E., Muleo R. (2017). The role of polyphenoloxidase, peroxidase, and ß-glucosidase in phenolics accumulation in *Olea europeaea* L. fruits under different water regimes. Front. Plant Sci..

[55] Põldma P., Moor U., Tõnutare T., Herodes K., Rebane R. (2013). Selenium treatment under field conditions affects mineral nutrition, yield and antioxidant properties of bulb onion (*Allium cepa* L.). Acta Sci. Pol. Hortorum Cultus.

[56] Kschonsek J., Wolfram T., Stöckl A., Böhm V. (2018). Polyphenolic compounds analysis of old and new apple cultivars and contribution pf polyphenolic profile to the in vitro antioxidant capacity. Antioxidants.

[57] Xu Y., Fan M., Ran J., Zhang T., Sun H., Dong M., Zhang Z., Zheng H. (2016). Variation in phenolic compounds and antioxidant activity in apple seeds of seven cultivars. Saudi J. Biol. Sci..

[58] Eichholz I., Rohn S., Gamm A., Beesk N., Herppich W.B., Kroh L.W., Ulrichs C., Huyskens-Keil S. (2012). UV-B-mediated flavonoid synthesis in white asparagus (*Asparagus officinalis* L.). Food Res. Int..

[59] Neugart S., Schreiner M. (2018). UVB and UVA as eustressors in horticultural and agricultural crops. Sci. Hortic..

[60] Scattino C., Castagna A., Neugart S., Chan H.M., Schreiner M., Crisosto C.H., Tonutti P., Ranieri A. (2014). Post-harvest UV-B irradiation induces changes of phenol contents and corresponding biosynthetic gene expression ion peaches and nectarines. Food Chem..

[61] Song Y., Yao Y., Zhai H., Du Y., Chen F., Wei S. (2007). Polyphenolic compound and the degree of browning in processing apple varieties. Agric. Sci. China.

[62] Allahveran A., Farokhzad A., Asghari M., Sarkhosh A. (2018). Foliar application of ascorbic and citric acids enhanced ‘Red Spur’ apple fruit quality, bioactive compounds and antioxidant activity. Physiol. Mol. Biol. Plants.

[63] Prior R.L., Wu X., Schaich K. (2005). Standardized methods for the determination of antioxidant capacity and phenolics in foods and dietary supplements. J. Agric. Food Chem..

[64] Wojdylo A., Oszmianski J., Laskowski P. (2008). Polyphenolic compounds and antioxidant activity of new and old apple varieties. J. Agric. Food Chem..

[65] Masuda I., Koike M., Nakashima S., Mizutani Y., Ozawa Y., Watanabe K., Sawada Y., Sugiyama H., Sugimoto A., Nojiri H. (2018). Apple procyanidins promote mitochnondrial biogenesis and proteoglycan biosynthesis in chondrocytes. Nature.

[66] Rana S., Bhushan S. (2016). Apple phenolics as nutraceuticals: Assessment, analysis and application. J. Food Sci. Technol..

[67] Dhyani P., Bahukhandi A., Rawat S., Bhatt I.D., Rawal R.S. (2018). Diversity of bioactive compounds and antioxidant activity in Delicious group in Western Himalaya. J. Food Sci. Technol..

[68] Zardo D.M., Silva K.M., Guyot S., Nogueira A. (2013). Phenolic profile and antioxidant capacity of the principal apples produced in Brazil. Int. J. Food Sci. Nutr..

[69] Csepregi K., Neugart S., Schreiner M., Hideg E. (2016). Comparative evaluation of total antioxidant capacities of plant polyphenols. Molecules.

[70] Pezzarossa B., Rosellini I., Malorgio F., Borghesi E., Tonutti P. Effects of selenium enrichment of tomato plants on ripe fruit metabolism and composition. Proceedings of the 7th International Postharvest Symposium.

[71] Riehle P., Vollmer M., Rohn S. (2013). Phenolic compounds in *Cistus incanus* herbal infusions—Antioxidant capacity and thermal stability during the brewing process. Food Res. Int..

[72] Zietz M., Weckmüller A., Schmidt S., Rohn S., Schreiner M., Krumbein A., Kroh L.W. (2010). Genotypic and climatic influence on the antioxidant activity of flavonoids in kale (*Brassica oleracea* var. *sabellica*). J. Agric. Food Chem..

[73] Fiol M., Weckmüller A., Neugart S., Schreiner M., Rohn S., Krumbein A., Kroh L.W. (2013). Thermal-induced changes of kale’s antioxidant activity analyzed by HPLC-UV/Vis-online-TEAC detection. Food Chem..

